# Muscle fibre size and myonuclear positioning in trained and aged humans

**DOI:** 10.1113/EP091567

**Published:** 2024-03-10

**Authors:** Edmund Battey, Yotam Levy, Ross D. Pollock, Jamie N. Pugh, Graeme L. Close, Michaeljohn Kalakoutis, Norman R. Lazarus, Stephen D. R. Harridge, Julien Ochala, Matthew J. Stroud

**Affiliations:** ^1^ Centre for Human & Applied Physiological Sciences, Faculty of Life Sciences & Medicine King's College London London UK; ^2^ British Heart Foundation Centre of Research Excellence, School of Cardiovascular Medicine and Sciences King's College London London UK; ^3^ Department of Biomedical Sciences, Faculty of Medical and Health Sciences University of Copenhagen Copenhagen Denmark; ^4^ School of Sport and Exercise Sciences, Tom Reilly Building, Byrom Street Liverpool John Moores University Liverpool UK; ^5^ Randall Centre for Cell and Molecular Biophysics, Faculty of Life Sciences & Medicine King's College London London UK

**Keywords:** ageing, cross‐sectional area, exercise, myonuclear domains, nuclei

## Abstract

Changes in myonuclear architecture and positioning are associated with exercise adaptations and ageing. However, data on the positioning and number of myonuclei following exercise are inconsistent. Additionally, whether myonuclear domains (MNDs; i.e., the theoretical volume of cytoplasm within which a myonucleus is responsible for transcribing DNA) and myonuclear positioning are altered with age remains unclear. The aim of this investigation was to investigate relationships between age and activity status and myonuclear domains and positioning. Vastus lateralis muscle biopsies from younger endurance‐trained (YT) and older endurance‐trained (OT) individuals were compared with age‐matched untrained counterparts (YU and OU; OU samples were acquired during surgical operation). Serial, optical *z*‐slices were acquired throughout isolated muscle fibres and analysed to give three‐dimensional coordinates for myonuclei and muscle fibre dimensions. The mean cross‐sectional area (CSA) of muscle fibres from OU individuals was 33%–53% smaller compared with the other groups. The number of nuclei relative to fibre CSA was 90% greater in OU compared with YU muscle fibres. Additionally, scaling of MND volume with fibre size was altered in older untrained individuals. The myonuclear arrangement, in contrast, was similar across groups. Fibre CSA and most myonuclear parameters were significantly associated with age in untrained individuals, but not in trained individuals. These data indicate that regular endurance exercise throughout the lifespan might better preserve the size of muscle fibres in older age and maintain the relationship between fibre size and MND volumes. Inactivity, however, might result in reduced muscle fibre size and altered myonuclear parameters.

## Abstract (highlights)

- **What is the central question of the study?**
  How do endurance exercise and the ageing process affect the positioning and number of myonuclei within muscle fibres?
- **What are the main findings and their importance?**
  There was reduced muscle fibre size, with smaller myonuclear domains and altered myonuclear domain scaling in older, untrained individuals. However, older trained individuals maintained similar muscle and myonuclear characteristics to younger trained individuals. This suggests that inactivity, rather than ageing, influences muscle fibre and myonuclear characteristics. These observations suggest that consistent endurance exercise over one's lifetime might help to preserve muscle fibre size and quality and myonuclear arrangement into old age.

## 1 INTRODUCTION

The myonuclear domain (MND) volume is the theoretical volume of cytoplasm for which a myonucleus is responsible (Allen et al., 1999). Changes in the architecture, number and organization of myonuclei and in MND size have been associated with muscle adaptation, including adaptation in response to exercise training, injury, disuse, disease and ageing (Bagley et al., 2023; Battey et al., 2023, 2020; Mohiuddin et al., 2019; Murach et al., 2020; Stroud et al., 2017). However, data on changes in MND size and myonuclear arrangement following exercise are inconsistent (Abreu et al., 2017; Verney et al., 2008). Additionally, there are conflicting reports regarding the relationship between MNDs and myonuclear arrangement with ageing (Cristea et al., 2010; Karlsen et al., 2015).

One possible explanation for the inconsistency of findings related to changes in MND sizes and the number and arrangement of myonuclei lies in the use of transverse cross‐sections and two‐dimensional muscle fibre analysis. Specifically, transverse cross‐sections of muscle biopsies from sedentary young, trained young, sedentary elderly and trained elderly individuals showed no differences in the number of myonuclei or MNDs between groups (Karlsen et al., 2015). Conversely, a three‐dimensional analysis of single muscle fibres revealed an age‐related decline in MND volume, reduced fibre size, variability of MND volume and myonuclear spacing (Cristea et al., 2010). However, physical activity levels were not accounted for in that study, preventing delineation between effects of inactivity and ageing. Thus, greater clarity on the relationship between the number and positioning of myonuclei, ageing and endurance exercise could be achieved by: (1) analysing longitudinal segments of muscle fibres in three dimensions; and (2) considering the influence of activity levels on age‐related observations.

We aimed to explore relationships between age and endurance exercise training status and the numbers and organization of myonuclei by analysing muscle fibres from younger trained (YT) and older trained (OT) individuals and their untrained counterparts (YU and OU, respectively). Muscle fibres were stained with 4',6‐diamidino‐2‐phenylindole (DAPI) and phalloidin, to mark DNA and F‐actin, respectively, followed by the acquisition of serial optical *z*‐slices through the fibre and quantification of three‐dimensional coordinates for myonuclei and muscle fibre dimensions. It was hypothesized that OU individuals would display aberrant myonuclear organization in comparison to YT, OU and OT individuals and that endurance‐trained individuals would have more evenly spaced nuclei in comparison to the other groups. Although we observed no significant association between ageing and myonuclear arrangement, the muscle fibres from OU individuals contained more myonuclei relative to fibre size and had a steeper, positive relationship between MNDs and fibre size. The number of nuclei per fibre was not lower in the OT and OU individuals, indicating that nuclei are preserved.

## 2 MATERIALS AND METHODS

### 2.1 Ethical approval

Before participation, written informed consent was obtained from all subjects. Procedures were approved by the Fulham Research Ethics Committee in London (12/LO/0457), Westminster Ethics Committee in London (12/LO/0457) or Liverpool John Moores Ethics Committee (H17SPS012) and conformed to the standards set by the *Declaration of Helsinki*, except for registration in a database. All human tissues were collected, stored and analysed in accordance with the Human Tissue Act (World Medical Association, 2013).

### 2.2 Characteristics of human participants

Four mixed‐sex groups were recruited to participate in this study (*n* = 6–8 per group). These groups were as follows: younger untrained healthy (YU; 33 ± 9.5 years), younger trained marathon runners (YT; 32 ± 5.4 years), older untrained individuals (OU; 79 ± 11.3 years) and older highly trained master cyclists (OT; 75.5 ± 3.2 years) (Table 1). Owing to sample availability, marathon runners rather than cyclists were used for the YT group.

**TABLE 1:** Participant characteristics.

Group	Age	Sex	Height (cm)	Weight (kg)	Body mass index (kg/m^2^)	Fibres analysed	Comment	Drug intake
Younger untrained	25	Female	164	55	20.4	16		
	27	Male	184	80	23.6	17		
	22	Male	178	75	23.7	18		
	32	Female	–	–	–	17		
	35	Female	–	–	–	17		
	42	Female	172	73	24.7	16		
	38	Female	158	48	19.2	17		
	44	Female	166	52	18.9	18		
Younger trained (marathon runners)	35	Male	182	71	21.4	14		
	32	Male	176	67	21.6	14		
	32	Female	170	64	22.1	13		
	22	Male	190	70	19.4	15		
	38	Male	170	68	23.5	12		
	34	Female	160	55	21.5	15		
Older untrained	59	Male	164	53	19.7	16	Osteoporosis	Perindopril, amlodipine, etidronate, adcal
	91	Female	–	–	–	18	Osteoporosis, diverticulitis with Hartmann's bowel operation	Furosemide, paracetamol, cholecalciferol, docusate
	77	Male	–	–	–	17	Osteoporosis	Paracetamol, tramadol, omeprazole, renetadine, mirapixin, procloperizone
	82	Female	152	57	24.7	13	Osteoporosis, hypertension, rheumatoid arthritis, type 2 diabetes	Metformin, denusomab, levothyroxine, allopurinol
	88	Female	156	64	26.3	17	Osteoporosis, breast cancer, left mastectomy in remission	Levothyroxine, atenolol
	77	Female	–	61	–	16	Osteoporosis, rheumatoid arthritis	Sulfasalazine
Older trained (master cyclists)	71	Male	180	83	25.7	18		
	76	Female	157	58	23.4	17		
	79	Female	159	53	21.1	12		
	75	Male	172	62	20.8	12		
	73	M	170	70	24.0	15		
	79	F	–	–	–	16		

The YU group was considered healthy, but not necessarily sedentary, given that two of the participants had been participating in low‐level recreational sport activities (fewer than two sessions per week) at the time of the study. Thus, the young cohort consisted of sedentary and low‐level physically active individuals. Participants were considered healthy if they met the criteria outlined by (Greig et al. 1994). The YT group consisted of trained recreational marathon runners (peak O_2_ uptake 56.7 ± 6.6 mL/kg/min, mean ± SD). In the YT group, the mean fastest running times (mean ± SD) in the previous 18 months over marathon, half marathon and 5 km were 204.5 ± 14.2, 88.5 ± 3.3 and 19.8 ± 1.3 min, respectively.

The OU group, used as a model for muscle disuse in old age, was a previously characterized cohort, who underwent dynamic hip screw insertion surgery (Kalakoutis et al., 2023). The patients completed a basic physical health questionnaire and were considered eligible if they did not suffer from neuromuscular disease. The hip fracture patients were recruited on the basis that they were likely to be frail individuals, and some had underlying health conditions (Table 1).

The OT group consisted of previously characterized individuals (Pollock et al., 2015, 2018) who were amateur master cyclists (maximal O_2_ uptake 36.76 ± 8.56 mL/kg/min, mean ± SD). Master cyclists were included if they were able to cycle 100 km in <6.5 h (males) or 60 km in <5.5 h (females). Participants must have had completed this distance within the specified time on two occasions in the 3 weeks before the date of participation in the study.

### 2.3 Muscle sampling and isolation of single muscle fibres

Vastus lateralis muscle samples from the YU, YT and OT participants were obtained using the same biopsy procedure. A portion of the mid‐thigh was shaved, and the skin was cleaned using chlorhexidine gluconate. Lignocaine (2%) was applied to the skin before making a 5 mm incision in the skin using a scalpel. A Bergström biopsy needle was inserted into the incision, and a biopsy of ∼200 mg was taken. Approximately 60 mg of the biopsy sample was then placed in a relaxing solution in a Petri dish on ice. Muscle tissue from the OU individuals was obtained during dynamic hip screw insertion surgery. During exposure of the hip during surgery, a portion of the vastus lateralis that becomes detached was placed into an ice‐cold relaxing solution. Muscle samples from biopsies and hip fracture surgeries were then prepared for skinned fibre experiments.

After excision, muscle samples (submerged in a relaxing solution in a Petri dish) were divided into bundles of ∼100 muscle fibres using forceps under a stereo microscope (Stemi 2000‐C, Zeiss) with a separate light source (Stereo CL 1500 ECO, Zeiss). The ends of the bundles were then tied onto glass capillary tubes using surgical silk (LOOK SP102) and stretched to ∼110% of the original length. These bundles were subsequently placed into 1.5 mL Eppendorf tubes containing skinning solution [relaxing solution with 50% (v/v) glycerol] at 4°C for 24 h to permeabilize the muscle fibres by disrupting the lipid bilayer of the sarcolemma, leaving myofilaments, intermediate filaments and the nuclear envelope intact (Frontera & Larsson, 1997; Konigsberg et al., 1975; Stienen, 2000; Wood et al., 1975). Samples were then treated in ascending gradients of sucrose dissolved in relaxing solution (0.5, 1.0, 1.5 and 2.0 M) for 30 min to prevent cryodamage (Frontera & Larsson, 1997). In a Petri dish containing 2 M sucrose, fibres were then removed from the glass capillary tubes before being placed in cryovials and snap‐frozen in liquid nitrogen.

For immunofluorescence experiments, muscle fibre bundles were placed in descending levels of sucrose dissolved in a relaxing solution, for 30 min in each solution (2.0, 1.5, 1.0, 0.5 and 0 M). Samples were transferred to the skinning solution at −20°C until the day of the experiment. To normalize orientation, single fibres were isolated from bundles using forceps and mounted on a half‐split grid for transmission electron microscopy glued to a coverslip. This was process was repeated for each fibre, such that an array of 9–11 fibres were mounted on the same platform.

### 2.4 Immunostaining, imaging and analysis of single muscle fibres

Muscle fibres were permeabilized in 0.2% Triton for 10 min and fixed in 4% Paraformaldehyde for 15 min. Muscle fibres were then incubated in a solution containing DAPI (Molecular Probes, D3571; 1:800) and Alexa Fluor 594 phalloidin (Invitrogen, A1238; 1; 1:100) for 1 h, to visualize myonuclei and actin, respectively. Finally, mounting medium (Fluoromount‐G) was added to the coverslip, and another 22 mm × 50 mm coverslip was placed carefully (to avoid bubbles) on top. Between each step of staining protocols, fibres were washed four times in PBS, leaving the fibres submersed in the final PBS wash for 5 min.

Images were acquired using a Zeiss Axiovert 200 confocal microscope equipped with a ×20 objective and CARV II confocal imager (BD Bioscience, San Jose, CA, USA) and an EXFO X‐Cite 120 mercury halide arc lamp. Fibres were aligned in parallel through clamping on a modified transmission electron microscopy grid, and the field of view was less than the length of the fibre segment, making fibre length consistent between conditions (∼330 μm). Damaged fibres were omitted from the analysis, and where there were clusters of nuclei, increased zoom and careful analysis of the orientation of nuclei through each *z*‐plane was carried out to identify the centroid of each nucleus. One hundred images were acquired, in 1 μm *z*‐increments, centred around approximately the middle of the fibre to ensure that the entire depth of the fibre was imaged.

Three‐dimensional visualization of muscle fibres and quantification of myonuclear arrangement was carried out using Metamorph software. The *z*‐stacks were imported, and each nucleus within the field of view (using the DAPI signal) and muscle fibre edges in *x*‐, *y*‐ and *z*‐planes (using the phalloidin signal) were recorded on Excel (linked to Metamorph). These data were analysed using a MATLAB script to obtain values for the MND (the volume of cytoplasm governed by each nucleus), order score (spatial distribution of nuclei), nearest neighbour distance (distance between nuclei) and fibre dimensions (Bruusgaard et al., 2003; Levy et al., 2018). Order score was calculated by generating a theoretical optimum distribution (MO) and a theoretical random distribution (MR) for each muscle fibre, based on the coordinates and numbers of corresponding myonuclei. This was compared with the experimental distribution (ME), and an order score was calculated with the expression: (ME − MR)/(MO − MR).

### 2.5 Statistical analyses

To analyse whether an overall significant difference was present between muscle fibres from the different human groups, Kruskal–Wallis tests were carried out, followed by Dunn's post hoc tests to specify between which groups differences existed. Mean values for each individual were used for two‐way ANOVA. For correlation analyses, a simple linear regression was performed to test whether slopes were significantly non‐zero, and non‐linear straight‐line regression analyses were performed to compare the slopes of different conditions. For all statistical tests, *P* < 0.05 indicated significance, and *P* ≤ 0.06 was considered a trend. Error bars in graphs represent the mean ± SD. All data were statistically analysed using Prism 9 (GraphPad).

## 3 RESULTS

**FIGURE 1:**
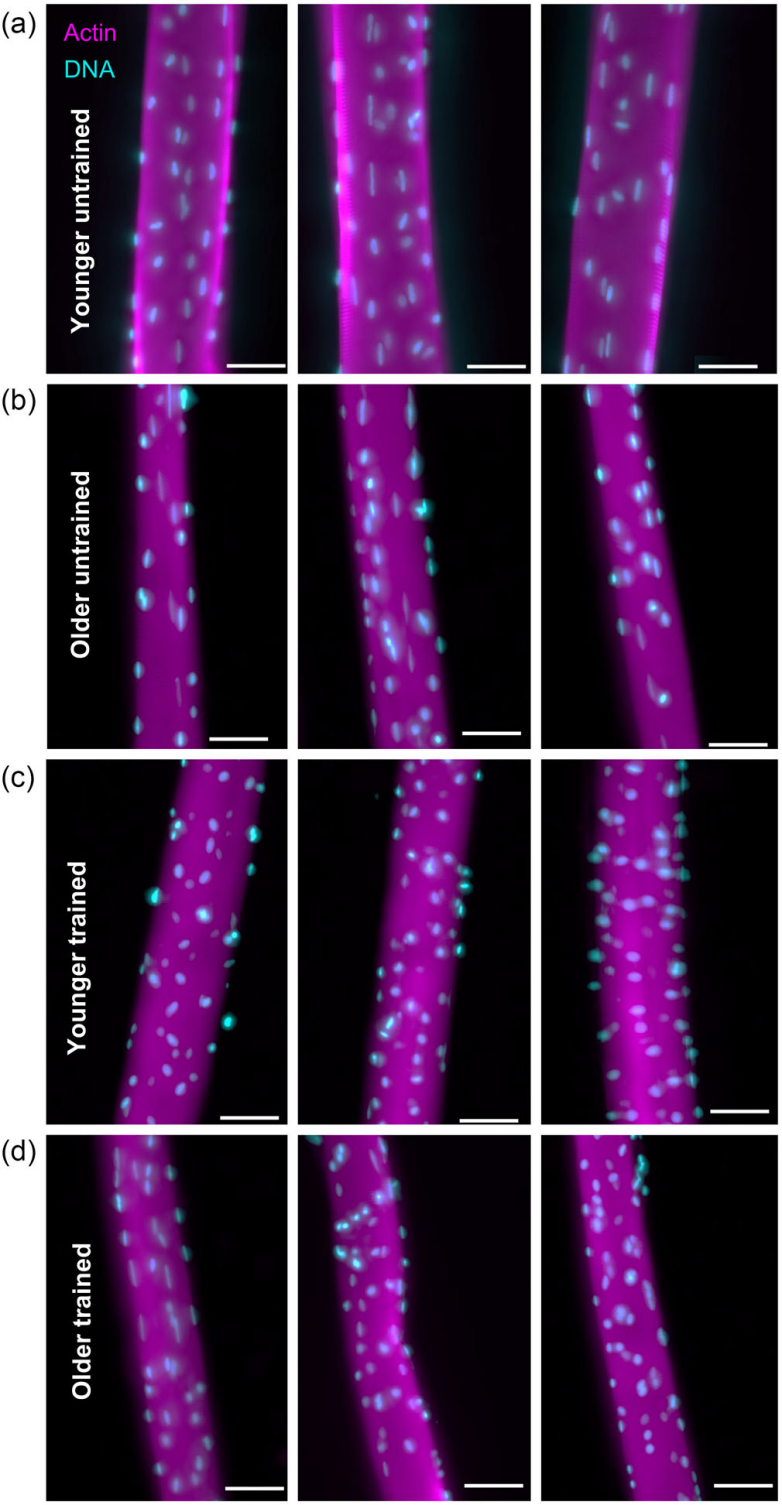
Representative images of myonuclear organization in younger and older untrained and endurance‐trained individuals. Representative images of vastus lateralis muscle fibres isolated from younger untrained (a), older untrained (b), younger trained (c) and older trained subjects (d) stained with DAPI (cyan) and phalloidin antibody (magenta) to visualize myonuclei and actin, respectively. Scale bars, 50 μm.

**FIGURE 2:**
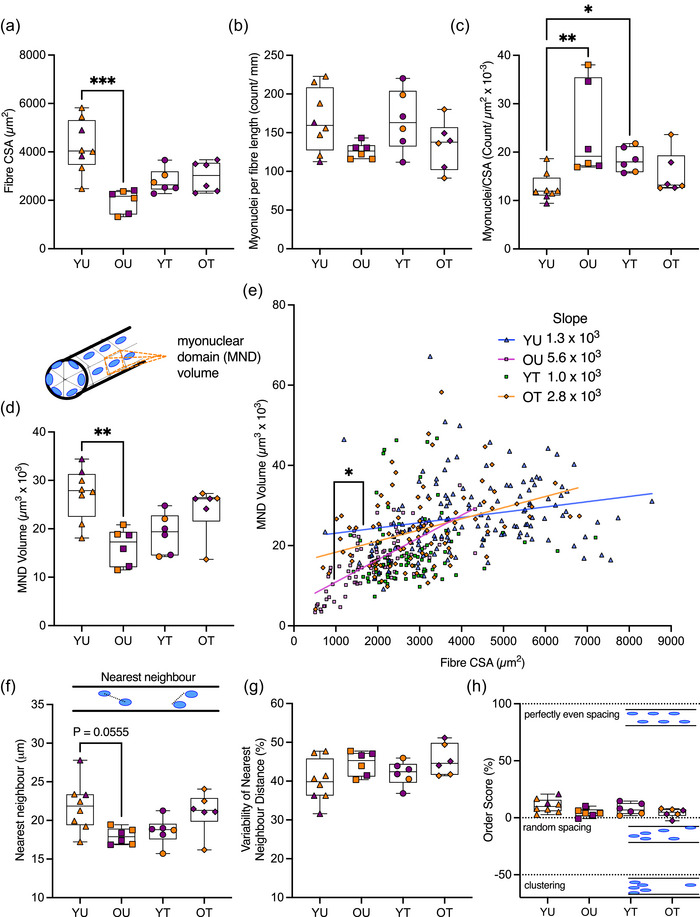
Muscle fibre size and number of myonuclei, myonuclear domains (MNDs) and organization in younger and older untrained and endurance‐trained individuals. (a–c) Comparisons of muscle fibre cross‐sectional area (CSA; in micrometres squared), myonuclei per fibre length and myonuclei normalized to fibre CSA in muscle fibres from younger untrained (YU), older untrained (OU), younger trained (YT) and older trained (OT) individuals. (d) Comparison of mean MND volumes across groups. Inset shows a cartoon of MND volume, with orange dotted lines highlighting the MND of a single myonucleus. (e) Relationship between muscle fibre CSA and MND volume (in micrometres cubed × 10^3^) in YU, OU, YT and OT individuals. Coloured symbols represent individual muscle fibres. A simple linear regression and straight‐line non‐linear regression were performed to quantify and compare gradients of slopes. Values of *R* ^2^: YU, 0.05; OU, 0.47; and OT, 0.15. There was no association between fibre CSA and MND volume in YT individuals, hence no regression line was plotted. (f, g) Comparison of nearest neighbour distances (in micrometres) and variability of nearest neighbour distances (as a percentage) across groups. (h) Comparison of mean order scores (as a percentage) across groups. Dotted lines represent clustered, random and perfectly even spacing of myonuclei. (a–d, f–h) Kruskal–Wallis tests were used for comparisons; box plots represent the median, interquartile range, minimum and maximum. Purple and orange symbols represent male and female individuals, respectively. ^*^ *P* < 0.05, ^**^ *P* < 0.01 and ^***^ *P* < 0.001.

To investigate the effects of ageing and exercise on the organization and number of myonuclei, MND volumes, myonuclear arrangement and fibre dimensions were compared in muscle fibres from YU, YT, OU and OT (136, 83, 96 and 88 fibres analysed per group, respectively; 403 fibres analysed in total). As expected, owing to the known effects of inactive ageing on muscle mass (Mckendry et al., 2018), the mean cross‐sectional area (CSA; in micrometres squared) of muscle fibres from OU was 33%–53% smaller compared with the other groups (OU, 1980 ± 1105; YU, 4238 ± 497; YT, 2941 ± 594; OT, 2985 ± 626; YU vs. OU. *P* < 0.05, Kruskal–Wallis test; Figures 1 and 2a). Although the number of nuclei per fibre length (as a count per millimetre) was unaffected by age or training status (indicating preservation of the number nuclei with age), the number of nuclei relative to fibre CSA was 90% and 45% greater in OU and YT compared with YU muscle fibres (OU, 24.1 ± 9.6; YT, 18.4 ± 2.6; YU, 12.7 ± 2.9; *P* < 0.05, Kruskal–Wallis test; Figure 2c).

In line with this, analysis of the volume of cytoplasm occupied by each myonucleus (MND; in micrometres cubed) revealed smaller mean MNDs in OU compared with YU fibres (16.3 ± 3.8 vs. 27.2 ± 5.4, respectively; *P* < 0.05, Kruskal–Wallis test; Figure 2d). Further analysis of the relationship between fibre CSA (in micrometres squared) and MND volumes (in micrometres cubed) showed a steeper slope in OU compared with YU fibres (*P* < 0.05; Figure 2e). This indicated that as fibre size increased, MNDs expanded to a greater extent in muscle fibres from OU compared with YU individuals (Figure 2e; *R* ^2^: YU, 0.05; OU, 0.47; OT, 0.15). There was no association between fibre CSA and MND volume in YT individuals.

In addition to the number of myonuclei and MNDs, myonuclear arrangement was quantified. Consistent with smaller MND volumes, there was a trend for a shorter mean distance between myonuclei (nearest neighbour, in micrometres) in OU compared with YU and in YT compared with YU fibres (*P* = 0.06, Kruskal–Wallis test; Figure 2f). Variability of nearest neighbour distance (as a percentage) was comparable between groups (Figure 2g). To gain further insight into the spatial arrangement of myonuclei, an order score (as a percentage) was generated to compute the distance between nuclei and fibre dimensions in *x*‐, *y*‐ and *z*‐planes (Bruusgaard et al., 2003; Levy et al., 2018). An order score of 100% indicates completely even spacing, 0% indicates random arrangement, and negative scores indicate clustering. Order scores were similar across all groups (YU, 10.7 ± 6.0; YT, 10.1 ± 5.3; OU, 3.8 ± 3.6; OT, 4.1 ± 3.8), indicating comparable myonuclear arrangement (Figure 2h).

**FIGURE 3:**
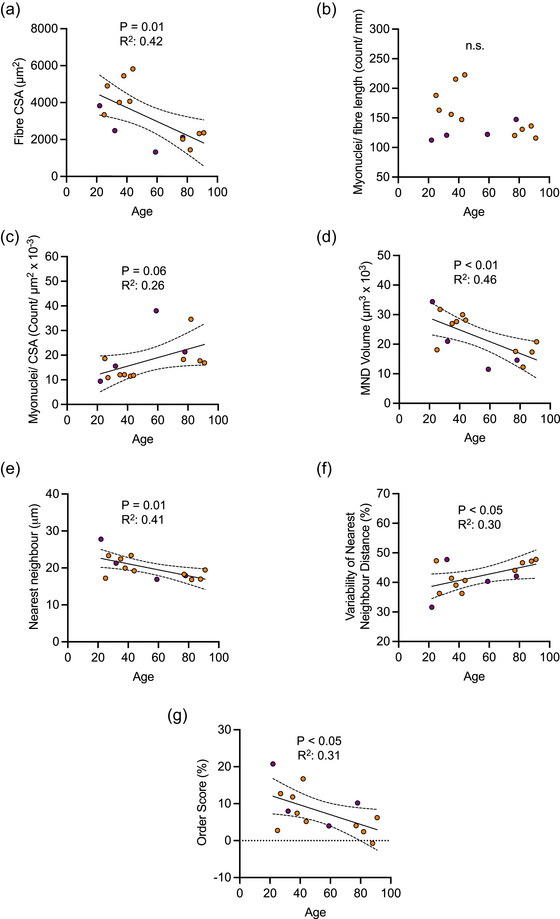
Associations between age and measured variables in untrained individuals: fibre size and myonuclear parameters. (a–g) Correlations between age for the following parameters: muscle fibre cross‐sectional area (CSA, in micrometres squared; a); number of nuclei per fibre length (as the count per millimetre) and per fibre CSA (as the count per micrometre squared^ ^× 10^−3^; b, c); myonuclear domain (MND) volume (in micrometres squared × 10^3^; d); mean nearest neighbour distance (in micrometres; e); variability of mean nearest neighbour distance (as a percentage; f); and mean order score (as a percentage; g). Symbols in purple represent male participants, and those in orange denote female participants.

**FIGURE 4:**
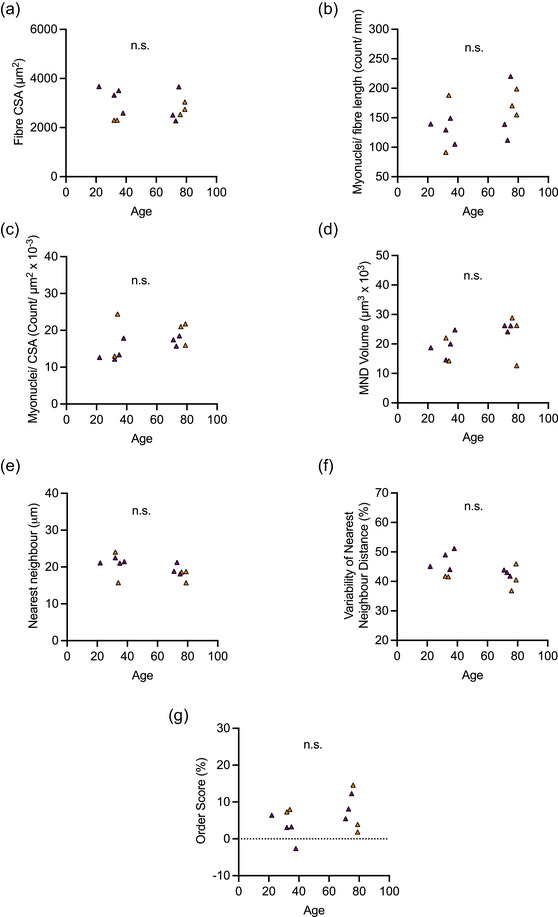
Associations between age and measured variables in trained individuals: fibre size and myonuclear parameters. (a–g) correlations between age the following parameters: muscle fibre cross‐sectional area (CSA, in micrometres squared; a); number of nuclei per fibre length (as the count per millimetre) and per fibre CSA (as the count per micrometre squared^ ^× 10^−3^; b, c); myonuclear domain (MND) volume (in micrometres squared × 10^3^; d); mean nearest neighbour distance (in micrometres; e); variability of mean nearest neighbour distance (as a percentage; f); and mean order score (as a percentage; g). Symbols in purple represent male participants, and those in orange denote female participants.

To examine the relationship between age and both fibre size and myonuclear parameters, we conducted linear regression analyses on the collected data, stratified by training status. In the untrained group, fibre CSA was negatively associated with age (*P* = 0.01, *R* ^2^ = 0.42; Figure 3a). The number of myonuclei/fibre length (in counts per millimetre) was not associated with age, whereas the number of myonuclei relative to fibre CSA trended towards a positive association with age (*P* = 0.06, *R* ^2^ = 0.26; Figure 3b,c). The MND volume (in micrometres cubed), variability of nearest neighbour distance (as a percentage) and order score (as a percentage) were negatively associated with age (*P* < 0.01, *R* ^2^ = 0.46; *P* = 0.01, *R* ^2^ = 0.41; and *P* < 0.05, *R* ^2^ = 0.31, respectively), whereas the nearest neighbour (in micrometres) was positively associated with age (*P* < 0.05, *R* ^2^ = 0.35; Figure 3d–g). In contrast to the untrained group, there were no associations between fibre CSA or myonuclear parameters and age in the trained group (Figure 4).

Together, these data show that muscle fibres from OU individuals are smaller than those from YU individuals and have more nuclei per fibre CSA and a greater expansion of the myonuclear domain as fibre size increases compared with muscle fibres from YU, YT and OT individuals. The number of myonuclei per fibre CSA was also greater in YT compared with YU individuals. Myonuclear arrangement, in contrast, was similar across groups. Correlation analyses support the suggestion that changes in fibre size and myonuclear parameters are related to inactivity rather than ageing per se.

## 4 DISCUSSION

A major problem in human ageing research and in skeletal muscle biology is to separate the effects of ageing from the negative effects of lifestyle factors that impact the ageing process (Distefano & Goodpaster, 2018; Lazarus & Harridge, 2017; Miljkovic et al., 2015), with one of the most documented being a lack of physical activity/exercise. It is widely recognized that the default position for humans is to be physically active and/or exercising regularly (Blair et al., 1989; Booth et al., 2012; Gries et al., 2018; Lazarus & Harridge, 2017; Pedersen & Saltin, 2015). In the absence of exercise, the ageing trajectory intersects with disease in and around the fifth to sixth decade of life. Thus, regular exercise helps to maintain physiological function with age, whereas a lack of exercise accelerates the decline in physiological function.

The aim of the present study was to determine the association between age and activity status regarding the number and organization of nuclei within single human skeletal muscle fibres. We did this by studying fibres obtained from four groups of individuals representing a spectrum of age and exercise status. The main findings were that in OU individuals, MNDs were smaller and the number of myonuclei relative to fibre size was greater than in YU individuals (Figure 2d). Additionally, MND volume in OU individuals expanded with increasing fibre size to a greater extent than in YU individuals (Figure 2e), with no association in the YT individuals between fibre size and MND.

Fibre size (Figure 2a) and myonuclear parameters (Figure 2b,c) were not significantly different in OT compared with OU individuals, which could be interpreted as exercise not having an impact. However, the lack of differences between YT and OT individuals coupled with the significant difference between YU and OU individuals (Figure 2a,c) indicate that in the absence of exercise there is an effect of age that is not present when exercise is performed. Furthermore, correlation analyses support the suggestion that changes in fibre size and myonuclear parameters are related to age when accompanied by inactivity (Figure 3) rather than with active ageing (Figure 4). Taken together, this suggests a complex interaction between age and exercise.

The higher number of myonuclei relative to fibre size in OU individuals indicates that myonuclei were retained during age‐associated disuse atrophy. If a net loss of myonuclei contributed to muscle atrophy, one would expect the number of myonuclei to decrease with fibre size. However, we observed that the number of myonuclei was maintained in smaller fibres, which is consistent with the observations of others (Malatesta et al., 2009; Snijders et al., 2020). These data support the concept that myonuclei are not lost with age or disuse atrophy (Gundersen, 2016; Gundersen et al., 2018). However, recent evidence demonstrating myonuclear loss and turnover suggests that new myonuclei could have been added to OU muscle fibres to maintain nuclear numbers (Kirby & Dupont‐Versteegden, 2022; Murach et al., 2020; Snijders et al., 2020). Loss of myonuclei has been demonstrated only in mice (Murach et al., 2020; Snijders et al., 2020); therefore, it is possible that this does not occur in humans and that the myonuclei in OU fibres have been retained throughout the lifespan. Nevertheless, we cannot rule out the possibility of this occurring in humans, and future longitudinal studies of disuse are needed to test this hypothesis. Our data highlight that the number of myonuclei alone is inadequate to maintain fibre size and suggest that other factors, such as exercise‐mediated gene expression and epigenetic changes, might be required to maintain muscle fibre size and quality (Sharples et al., 2018; Widmann et al., 2019). Additionally, the spatial transcriptional and translational control of protein synthesis might be important in the adaptive response to endurance training (Bagley et al., 2023; Figueiredo & McCarthy, 2019). Some mRNAs might remain localized around nuclei, making ribosomes a limiting factor for protein synthesis in this case, whereas some proteins might be able to travel far from the nucleus of its mRNA origin (Roman et al., 2021). An alternative perspective is that during a period of disuse, muscle fibres adapt by becoming smaller, because the ability to produce force is not required; during this period, myonuclei remain, resulting in a greater number of myonuclei relative to fibre size.

We observed that endurance‐trained individuals did not have a higher number of myonuclei compared with untrained individuals, suggesting that myonuclear accretion might not be a major endurance training adaptation. This is in line with previous evidence showing no increase in the number of myonuclei with endurance training in humans (Charifi et al., 2003; Snijders et al., 2011; Verney et al., 2008), despite reports of an increased number of satellite cells and their activation after endurance training (Appell et al., 1988; Charifi et al., 2003; Verney et al., 2008). These results also demonstrate that lifelong aerobic cycling exercise in older age does not affect the number of myonuclei in single muscle fibres, supporting the results of a previous study in trained runners (Karlsen et al., 2015). Interestingly, we observed reduced MND volume in YT compared with YU individuals, but this might be explained by the ∼25% smaller mean fibre size (not significant) resulting in a relative decrease in nuclear spacing.

Collectively, these data suggest that, unlike the increase observed after resistance training (Petrella et al., 2008), endurance training is not associated with a substantial increase in the number of myonuclei. Thus, it is tempting to speculate that with endurance training, myonuclei adapt their epigenetic and transcriptional landscapes versus nuclear accretion as observed in fibres from resistance‐trained individuals.

No changes in myonuclear organization were observed with age, in contrast to a previous report that showed higher variability of myonuclear positioning in type I fibres of older participants (Cristea et al., 2010). In the present investigation, some clustering and myonuclear disorganization was observed in all groups, but these had less overall weight on the average values obtained. Thus, it appears that myonuclear organization might be altered in some fibres in older groups, but not significantly more than other groups. A methodological limitation of the present study is that, in the interest of increasing the throughput of staining and imaging to acquire data on sufficient muscle fibres for statistical analyses, fibre type co‐staining was not performed. Thus, potential fibre type‐related changes in MND volume were not accounted for (Liu et al., 2009). This additional fibre type‐specific analysis would be required to address directly whether differences in fibre type proportions across groups affected the number and arrangement of myonuclei. Future studies should include the gold‐standard myosin heavy chain fibre typing when considering myonuclear characteristics in response to ageing and exercise. This fibre typing can be done with fluorescence labelling co‐staining, using SDS–PAGE using a fibre piece clipped before imaging (Machek et al., 2021) or using a newer ‘dot blotting’ technique on a small piece of fibre clipped before imaging (Christiansen et al., 2019). A chemical skinning procedure was used to remove non‐muscle cells in order that MNDs were analysed and not domains of nuclei from non‐muscle cells. However, the possibility that some strongly adhered cells might remain after chemical skinning cannot be ruled out completely, which might affect the number of myonuclei and the myonuclear organization parameters measured.

We observed no difference in mean fibre CSA in the older trained group compared with the other groups, in line with a previous study on lifelong strength‐trained, endurance‐trained and untrained elderly individuals (Aagaard et al., 2007). However, these findings are in contrast to a recent study on lifelong endurance exercisers and a previous study on younger endurance athletes and age‐matched control subjects (Grosicki et al., 2021; Harber & Trappe, 2008). These disparities might be explained by the pooling of fibre types, with lifelong endurance exercisers from this cohort having a high distribution of smaller type I fibres (Pollock et al., 2018). Additionally, the YU group studied might have had a greater proportion of type IIA fibres compared with the OT group, as recently shown by Kalakoutis et al. (2023). Another possible explanation for the greater fibre CSA of some individuals in the YU group compared with the trained groups is that smaller fibres in endurance‐trained individuals could be an adaptive response; efficiency is key for endurance performance, and added muscle mass might increase the weight carried at the expense of efficiency (Coggan et al., 1992; Hughes et al., 2018; Joyner & Coyle, 2008). Additionally, increased muscle fibre size might increase the diffusion distance between capillaries and mitochondria. This could increase the time it takes for oxygen to reach mitochondria, which might limit the rate of ATP production through aerobic metabolism, thereby compromising endurance performance. Lifelong endurance training can attenuate age‐related muscle atrophy, which could explain the lack of significant difference in fibre CSA between YU and OT individuals and the significantly smaller CSA of muscle fibres from OU compared with YU individuals (Mckendry et al., 2018; Wroblewski et al., 2011).

Our results on the number of myonuclei and their organization also need to be interpreted in the context of our previous findings on these fibres regarding the shape of individual nuclei (Battey et al., 2023). Here, we observed that fibres from both young and old untrained individuals were less spherical, and nuclei from older untrained individuals were more deformable and contained a thinner nuclear lamina than those from the older trained individuals. However, in contrast to the known adaptability of inactive young muscle fibres to exercise training, adaptation to exercise is more limited in older muscle [e.g., anabolic resistance to exercise (Kumar et al., 2009) and feeding (Cuthbertson et al., 2005)]. This suggests that despite exhibiting some similar nuclear organization features to YU, YT and OT fibres, the fibres from OU individuals are compromised by an interaction of ageing and disuse, such as altered nuclear shape and mechanics (Battey et al., 2023).

We acknowledge that although we attempted to create a model in which age and exercise interactions could be studied, there are some limitations, given the donors from whom fibres were collected. These include: (1) relatively small sample sizes that were not equally balanced between groups for sex; (2) although endurance exercisers were studied, these were runners in the YT group, whereas they were cyclists in the OT group; (3) the OU group were not simply healthy older individuals who were less active than the OT group, but older patients undergoing hip surgery who were also on different medications (Table 1), with the effects of this on muscle not being fully understood; and (4) the YU group, although untrained had varying levels of physical activity, perhaps explaining, in part, the greater heterogeneity of fibre size in this group. Additionally, it is important to consider that the possibility of the difference in muscle sampling method between the OU group and other groups contributing to the observations cannot be ruled out (muscle acquired during surgical procedures compared with a vastus lateralis biopsy using a Bergström needle).

With these caveats in mind, the data do, however, suggest that regular endurance exercise throughout the lifespan helps to preserve the size and MND volumes of single muscle fibres. Young individuals who remain inactive are likely to be on a trajectory towards longer‐term pathology, which might include reduced muscle fibre size despite maintaining the number of myonuclei. Furthermore, in addition to the number of myonuclei and their organization, myonuclear structure and mechanics (Battey et al., 2023) are also likely to contribute to both reduced muscle fibre size and other transcriptionally regulated aspects of muscle associated with inactive ageing. Ideally, longitudinal studies comparing untrained and trained individuals who are more tightly defined will shed greater light on the interaction between ageing and inactivity processes in muscle.

## AUTHOR CONTRIBUTIONS

Matthew J. Stroud, Julien Ochala and Edmund Battey contributed to the conception of the work. Edmund Battey, Julien Ochala and Yotam Levy designed experiments. Edmund Battey, Yotam Levy and Julien Ochala performed experiments. Edmund Battey and Yotam Levy completed the formal analysis of the data. Edmund Battey interpreted the data and completed data visualization. Ross D. Pollock, Michaeljohn Kalakoutis, Jamie N. Pugh, Graeme L. Close, Norman R. Lazarus, Stephen D. R. Harridge and Julien Ochala recruited human participants and collected human muscle biopsy samples. Edmund Battey wrote the first draft of the manuscript. All authors approved the final version of the manuscript and agree to be accountable for all aspects of the work in ensuring that questions related to the accuracy or integrity of any part of the work are appropriately investigated and resolved. All persons designated as authors qualify for authorship, and all those who qualify for authorship are listed.

## CONFLICT OF INTEREST

None declared.

## Data Availability

Individual data points (*n* ≤ 30) are included in the figures. Data from the study will be made available upon reasonable request.
